# Increased level of nucleolin confers to aggressive tumor progression and poor prognosis in patients with hepatocellular carcinoma after hepatectomy

**DOI:** 10.1186/s13000-014-0175-y

**Published:** 2014-09-18

**Authors:** XiaoDong Guo, Lu Xiong, Lingxiang Yu, Ruisheng Li, ZhaoHai Wang, Bo Ren, JingHui Dong, Boan Li, Dadong Wang

**Affiliations:** 302 Hospital of PLA, Beijing, 100039 China; Beijing Institute of Radiation Medicine, Beijing, 100850 China; Department of Hepatobiliary and Pancreaticosplenic Surgery, the First Affiliated Hospital of General Hospital of PLA, Beijing, 100048 China

**Keywords:** Hepatocellular carcinoma, Nucleolin, Expression, Tumor progression, Prognosis

## Abstract

**Background:**

Nucleolin, as a multifunctional protein, has been demonstrated to play an oncogenic role in human hepatocellular carcinoma (HCC). The aim of this study was to investigate the expression pattern of nucleolin in HCC and determine its correlation with tumor progression and prognosis.

**Methods:**

Nucleolin expression at both mRNA and protein levels in HCC and adjacent nonneoplastic tissues were respectively detected by quantitative real time polymerase chain reaction (Q-PCR), immunohistochemistry and western blotting.

**Results:**

Nucleolin expression, at both mRNA and protein levels, was significantly higher in HCC tissues than in the adjacent nonneoplastic tissues (both P < 0.001). In addition, the elevated nucleolin expression was markedly correlated with advanced tumor stage (P = 0.001), high tumor grade (P = 0.02) and serum AFP level (P = 0.008). Moreover, HCC patients with high nucleolin expression had shorter 5-year disease-free survival and shorter 5-year overall survival than those with low expression (both P < 0.001). Furthermore, the Cox proportional hazards model showed that nucleolin expression was an independent poor prognostic factor for both 5-year disease-free survival (hazards ratio [HR] = 3.696, 95% confidence interval [CI] = 1.662-8.138, P = 0.01) and 5-year overall survival (HR = 3.872, CI = 1.681-8.392, P = 0.01) in HCC.

**Conclusion:**

These results showed that the markedly and consistently increasing expression of nucleolin may be associated with aggressive characteristics of HCC, and implied that nucleolin expression may serve as a promising biochemical marker for predicting the clinical outcome of patients with this malignancy.

**Virtual Slides:**

The virtual slide(s) for this article can be found here: http://www.diagnosticpathology.diagnomx.eu/vs/13000_2014_175.

## Background

Hepatocellular carcinoma (HCC), accounts for approximately 90% of liver cancers, is the third cause of cancer mortality worldwide, with 700,000 new cases annually [1]. Nearly 85% cases with HCC occur in developing countries of East Asia and sub-Saharan Africa [2]. In China, HCC is the second leading cause of cancer-related death among men. Its five-year postoperative survival rate is only 30% to 40% [3]. As a highly aggressive solid tumor, HCC is characterized by fast infiltrating growth, early metastasis, high-grade malignancy, and poor prognosis. Currently, hepatic resection is the first choice for patients suffering with HCC, however, the long-term survival remains unsatisfactory mainly due to a high incidence of postoperative metastasis and recurrence, and the high resistance of HCC to chemotherapy [4]. Accumulating studies have demonstrated that the progression of HCC is a complicate process which is associated with environmental factors (such as infection with HBV and alcoholic liver disease) and genetic/epigenetic alterations [5]. However, the molecular mechanism of the tumor development and progression in HCC has not been fully elucidated. Thus, it is extremely necessary to illustrate the mechanisms underlying hepatocarcinogenesis and the process of tumor invasion and tumor metastasis, and further identify efficient molecular markers for early diagnosis and prognosis as well as valuable therapeutic targets.

Nucleolin is a multifunctional and mobile protein which can shuttle among the nucleolus, nucleoplasm, cytoplasm, and cytoplasmic membrane [6]. It is originally identified by Orrick et al. [7] in 1973, and then, the same protein was extracted from ovary cells of Chinese hamster, and several other eukaryotic cells, including human, rat, mouse, and chicken [8]. Nucleolin has an apparent molecular mass of 100–110 kDa as determined by SDS-PAGE and a calculated molecular mass of 76–77 kDa as predicted by the amino acid sequence. This difference may be due to posttranslational modifications and a high content of negatively charged amino acids in the N-terminal part of nucleolin [9]. Functionally, it is implicated in many aspects of DNA metabolism, and participates extensively in RNA regulatory mechanisms, including transcription, ribosome assembly, mRNA stability and translation, and microRNA processing [10]. Nucleolin is also involved in pathological processes, particularly cancer. It is overexpressed in highly proliferative cells, especially in several malignant cells, including neuroblastoma, cutaneous melanoma, pediatric intracranial ependymoma, breast cancer, lung cancer, colon cancer [11-16]. Nucleolin functions as a oncogene and regulates the abilities of cancer cells to grow and proliferate, overcome senescence, evade apoptosis and the immune system, invade and metastasize other tissues and promote angiogenesis. To our interests, Bao et al. [17] identified nucleolin as a candidate biomarker for diagnosis of HCC by quantitative proteomic analysis of a paired human liver healthy versus carcinoma cell lines with the same genetic background; Meng et al. [18] further found that the downregulation of nucleolin expression may inhibit the growth of HCC cells in vitro. These previous studies suggest that nucleolin plays a role in HCC. However, its clinical significance remains unclear. Therefore, the aim of the current study was to investigate the expression pattern of nucleolin in HCC and determine its correlation with tumor progression and prognosis.

## Methods

### Patients and tissue samples

The study was approved by the Research Ethics Committee of 302nd Hospital of PLA, Beijing, China. Informed consent was obtained from all of the patients. All specimens were handled and made anonymous according to the ethical and legal standards.

A total of 130 patients with primary HCC who underwent a curative liver resection at the 302nd Hospital of PLA, Beijing, China, were included in this retrospective study. Tissues used in the study were retrieved from the tissue bank of the Department of Pathology in the 302nd Hospital of PLA. These patients were diagnosed as HCC between March 2001 and February 2006. None of the patients recruited in this study had chemotherapy or radiotherapy before the surgery. HCC diagnosis was based on World Health Organization (WHO) criteria [19]. Tumor differentiation was defined according to the Edmondson grading system [20]. Liver function was assessed using the Child-Pugh scoring system [21]. Tumor staging was determined according to the sixth edition of the tumor-node-metastasis (TNM) classification of the International Union against Cancer [22]. The clinicopathological features of 130 patients are summarized in Table 1. In addition, 30 matched HCC specimens (5 TNM stage I, 8 TNM stage II, 12 TNM stage III, and 5 TNM stage IV) and adjacent nonneoplastic liver tissues were collected between April 2008 and March 2010, snap-frozen in liquid nitrogen and stored at −80°C following surgery for real-time quantitative RT-PCR assay and western blot analysis.

**Table 1:** **Clinicopathological features and the expression of nucleolin in 130 hepatocellular carcinoma patients**

**Clinicopathological features**	**Case**	**Nucleolin expression frequency (n,%)**		**P**
		**High**	**Low**	
**Age (years)**				
≤50	72	40 (55.56)	32 (44.44)	NS
>50	58	28 (48.28)	20 (51.72)	
**Gender**				
Male	96	58 (60.42)	42 (39.58)	NS
Female	34	20 (58.82)	14 (41.18)	
**Serum AFP**				
Positive	72	58 (80.56)	14 (19.44)	0.008
Negative	58	20 (34.48)	38 (65.52)	
**Tumor stage**				
T1	23	0 (0)	23 (100.00)	0.001
T2	40	23 (57.50)	17 (42.50)	
T3	52	40 (76.92)	12 (23.08)	
T4	15	15 (100.00)	0 (0)	
**Tumor grade**				
G1	31	12 (38.71)	19 (61.29)	0.02
G2	76	48 (63.16)	28 (36.84)	
G3	23	18 (78.26)	5 (21.74)	
**Growth pattern**				
Trabecular	101	59 (58.42)	42 (41.58)	NS
Nontrabecular	29	19 (65.52)	10 (34.48)	
**Cirrhosis**				
Yes	86	50 (58.14)	36 (41.86)	NS
No	44	28 (63.64)	16 (36.46)	
**Underlying liver disease**				
Alcoholic	25	10 (40.00)	15 (60.00)	NS
Hepatitis B	49	28 (57.14)	21 (42.86)	
Hepatitis C	35	28 (80.00)	7 (20.00)	
Unknown	21	12 (57.14)	9 (42.86)	

Note: ‘NS’ refers to the differences among groups have no statistical significance.

The median follow-up period of 130 patients with primary HCC was 8.6 years. Postoperative surveillance included routine clinical and laboratory examinations every third month, computed tomography scans of the abdomen, and radiographs of the chest every third month. After 5 years, the examination interval was extended to 12 months.

### Quantitative RT-PCR

To measure the mRNA expression levels of nucleolin, total RNA was extracted from frozen liver tissues using TriZol reagent (Invitrogen) following the manufacturer’s instructions. Two micrograms of total RNA was subjected to reverse transcription to synthesize cDNA using the ProtoScript M-MuLV Taq RT-PCR Kit (New England Biolabs), according to the manufacture’s instruction, followed by real-time PCR using the TransStart Green qPCR SuperMix (TransGen Biotech). The primer sequences of nucleolin were forward primer, 5′- GCA CTT GGA GTG GTG AAT CAA A-3′, reverse primer, 5′- AAA TGC ATA CCC TTT AGA TTT GCC-3′. The transcription of GAPDH was used as an internal control for normalization. nucleolin expression levels were calculated relative to GAPDH using the delta-delta cycle threshold method [23].

### Western blot

The Western blot protocol and semiquantitative analysis were carried out following the protocol of Xu et al. [24]. nucleolin antibody (mouse monoclonal antibody, dilution 1:100, 4E2, #ab13541, Abcam Inc., Cambridge, UK) was used, and GAPDH antibody (CW0266, dilution 1:1,000, CoWin Biotech) was used as internal control.

### Immunohistochemistry analysis

Immunohistochemical staining was carried out following the protocol of our previous study [25-27]. The primary antibody against nucleolin: mouse monoclonal antibody (4E2, #ab13541, Abcam Inc., Cambridge, UK), dilution 1:100. Secondary antibody for the detection of primary antibody: anti-goat IgG (#sc-2028, Santa Cruz Biotechnology, Inc. USA). The negative controls were processed in a similar manner with PBS instead of primary antibody. The positive nucleolin expression confirmed by western blotting was used as positive controls for immunostaining.

Following a hematoxylin counterstaining, immunostaining was scored by two independent experienced pathologists, who were blinded to the clinicopathological parameters and clinical outcomes of the patients. The scores of the two pathologists were compared and any discrepant scores were reconciled through re-examining the stainings by both pathologists to achieve a consensus score. The number of positive-staining cells showing immunoreactivity in the nucleus, cytoplasm and/or cytoplasmic membrane for nucleolin in ten representative microscopic fields was counted and the percentage of positive cells was calculated. The percentage scoring of immunoreactive tumor cells was as follows: 0 (0%), 1 (1-10%), 2 (11-50%) and 3 (>50%). The staining intensity was visually scored and stratified as follows: 0 (negative), 1 (weak), 2 (moderate) and 3 (strong). A final score was obtained for each case by multiplying the percentage and the intensity score. Therefore, tumors with a multiplied score of less than 5.62 (median of total scores for nucleolin) were considered to be low nucleolin expressers; all other scores were considered to be high nucleolin expressers.

### Statistical analysis

The software of SPSS version13.0 for Windows (SPSS Inc, IL, USA) and SAS 9.1 (SAS Institute, Cary, NC) was used for statistical analysis. Fisher’s exact test [28] and the X^2^ test [29] were performed to assess associations between nucleolin expression and clinicopathological parameters. The Kaplan-Meier method [30] was used for survival analysis, and differences in survival were estimated using the log-rank test [31]. A multivariate survival analysis was performed for all parameters that were significant in the univariate analyses using the Cox regression model [32]. Differences were considered statistically significant when *P* was less than 0.05.

## Results

### Increased expression of nucleolin mRNA and protein in HCC

**Figure 1:**
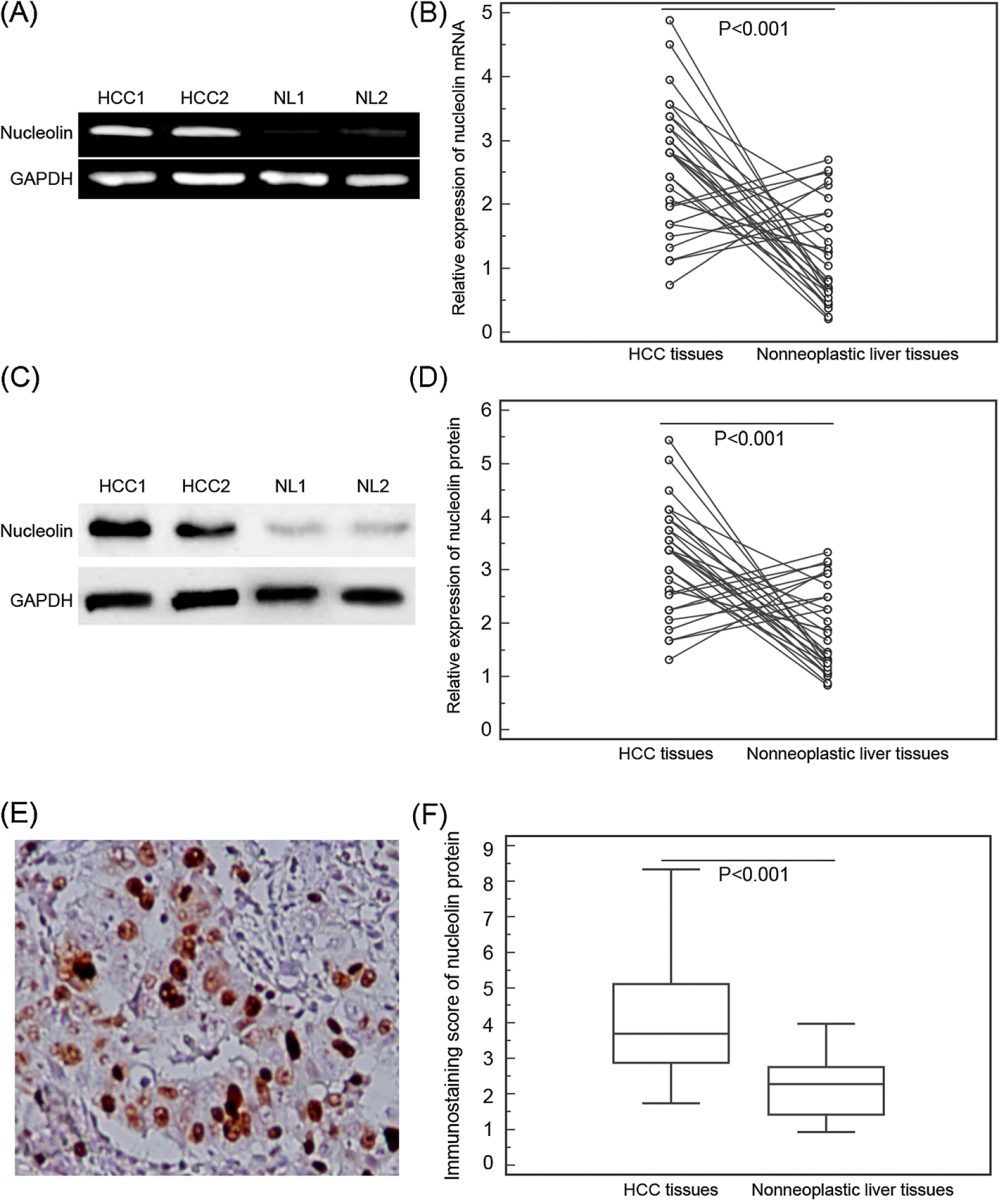
**Nucleolin mRNA and protein expression in hepatocellular carcinoma (HCC) and adjacent nonneoplastic liver tissues. (A)** Representative graph of nucleolin mRNA levels in HCC tissues and adjacent nonneoplastic liver tissues (NL). **(B)** Quantitative RT-PCR assay showed significantly decreased nucleolin mRNA level in HCC tissues compared with adjacent nonneoplastic liver tissues (P < 0.001). **(C)** Representative Western blotting of nucleolin protein levels in HCC tissues and adjacent nonneoplastic liver tissues (NL). **(D)** Semiquantitative Western blotting showed that the expression levels of nucleolin protein were significantly lower than those in adjacent nonneoplastic liver tissues (P < 0.001). GAPDH was used as internal control. Means, standard deviation (SD), and P values were given (T test). **(E)**, nucleolin positive staining was indicated by numerous yellowish granules in the nucleus, cytoplasm and/or cytoplasmic membrane of HCC cells (Original magnification × 400). **(F)** Semiquantitative immunoreactive scores showed that the expression levels of nucleolin protein were significantly lower than those in adjacent nonneoplastic liver tissues (P < 0.01).

Quantitative RT-PCR was performed to compare the expression level of nucleolin mRNA between 30 matched tumor-normal specimens of HCC. We found that 22 of 30 patients (73.33%) had higher nucleolin mRNA expression in HCC tissues than in adjacent nonneoplastic liver tissues. Statistical analysis showed that the relative expression of nucleolin mRNA in HCC tissues was significantly higher than in the adjacent nonneoplastic liver tissues (2.61 ± 1.00 vs. 1.22 ± 0.76, P < 0.001; Figure 1A and B). In addition, nucleolin expression at protein level in HCC and adjacent nonneoplastic liver tissues were also measured by Western blotting. Similar with the quantitative RT-PCR results, the expression levels of nucleolin protein in HCC tissues were markedly higher than those in the adjacent nonneoplastic liver tissues (3.17 ± 1.06 vs. 1.89 ± 0.79, P < 0.001; Figure 1C and D).

In order to confirm the subcellular localization of nucleolin protein in HCC tissues, immunohistochemical analysis was performed using 130 matched tumor-normal specimens of HCC. The immunostaining for nucleolin was observed in the nucleus, cytoplasm and/or cytoplasmic membrane of HCC cells (Figure 1E). The immunostaining scores of nucleolin protein in HCC tissues were significantly higher than those in adjacent nonneoplastic liver tissues (5.19 ± 3.17 vs. 2.43 ± 1.34, P < 0.001; Figure 1F). In 130 HCC tissues, 78 (75/130, 60.00%) showed high nucleolin expression, while 52 (40.00%, 52/130) displayed low nucleolin expression.

### Association of nucleolin expression with the clinicopathological features of HCC

To determine whether nucleolin protein expression levels are indicative of the state of HCC progression, we analyzed the association between nucleolin immunostaining scores and tumor stage, tumor grade, serum AFP level, presence of cirrhosis, underlying liver disease including alcohol abuse, viral hepatitis B and C, gender, and age. As summarized in Table 1, the statistically significant correlations were observed between nucleolin expression and tumor stage, tumor grade and serum AFP level. The high nucleolin expression more frequently occurred in HCC tissues with advanced tumor stage (P = 0.001), high tumor grade (P = 0.02) and high serum AFP level (P = 0.008) than those with early tumor stage, low tumor grade and low serum AFP level. In addition, nucleolin expression was not statistically associated with patient age or gender, or several of the classic markers for HCC progression, including the presence of cirrhosis and underlying liver diseases.

### Prognostic values of nucleolin expression in HCC

**Figure 2:**
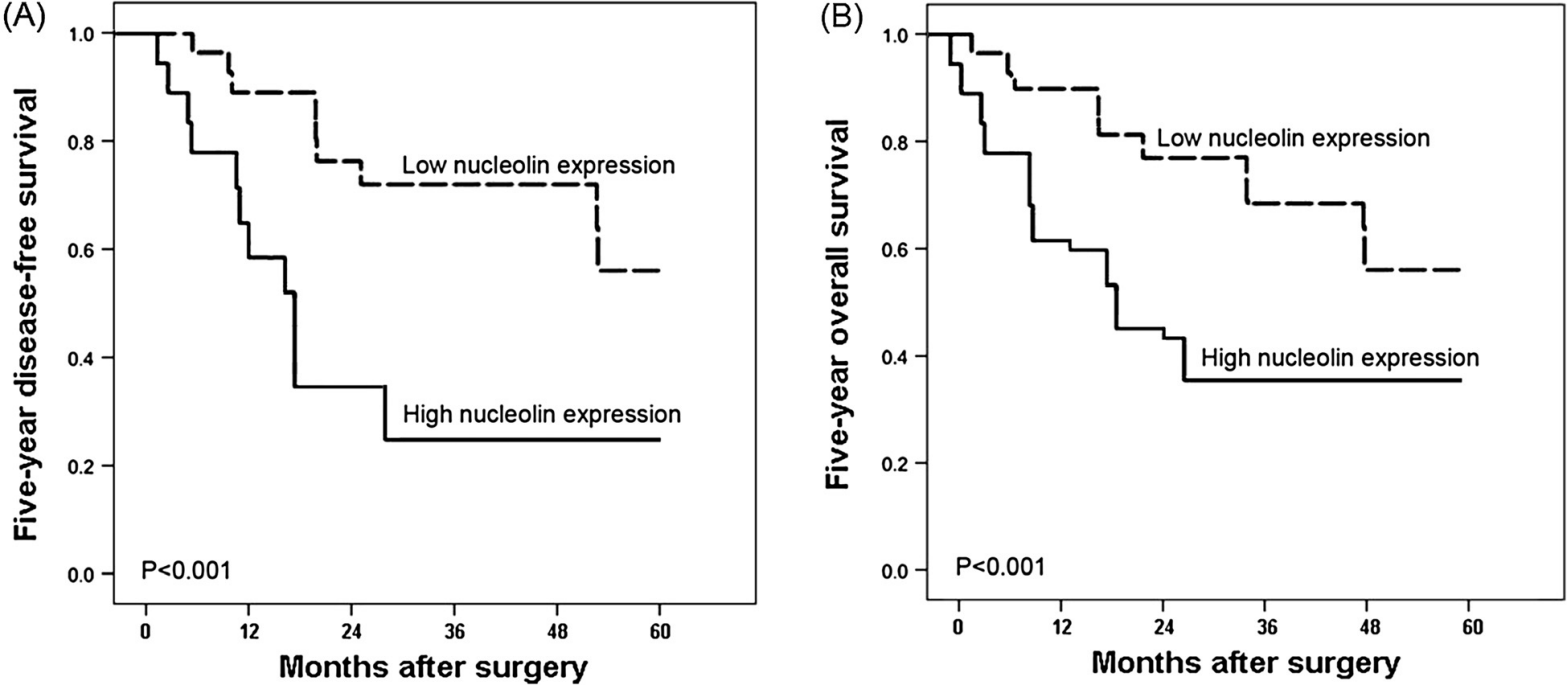
**Kaplan-Meier survival curves for nucleolin expression in hepatocellular carcinoma (HCC) patients.** HCC patients with high nucleolin expression had shorter 5-year disease-free survival **(A)** and shorter 5-year overall survival **(B)** than those with low expression (both P < 0.001).

Five-year disease-free survival was observed in 30 (23.08%) patients, whereas in 100 (76.92%) patients, disease recurred, and 88 (67.69%) even died during a 5-year follow-up period. We found a trend that 5-year disease-free survival in the group with high nucleolin expression was significantly shorter than that in the group with low expression (P < 0.001, log-rank test; Figure 2A). Additionally, the Kaplan-Meier plot of 5-year overall survival curves stratified by nucleolin expression was shown in Figure 2B. A significant relationship was also found between nucleolin expression and 5-year overall survival (P < 0.001, log-rank test, Figure 2B). Furthermore, the Cox proportional hazards model showed that nucleolin expression was an independent poor prognostic factor for both 5-year disease-free survival (hazards ratio [HR] = 3.696, 95% confidence interval [CI] = 1.662-8.138, P = 0.01, Table 2) and 5-year overall survival (HR = 3.872, CI = 1.681-8.392, P = 0.01, Table 2) in HCC.

**Table 2:** **Multivariate survival analysis of five-year overall and disease-free survival in 130 patients with hepatocellular carcinoma**

**Features**	**Five-year overall survival**			**Five-year disease-free survival**		
	**HR**	**95% CI**	**P**	**HR**	**95% CI**	**P**
**Serum AFP**	1.931	0.685-4.056	0.063	1.953	0.615-4.273	0.062
**Tumor stage**	2.879	1.366-5.196	0.009	2.686	1.386-6.009	0.01
**Tumor grade**	1. 563	0.609-4.088	0.081	1.551	0.607-4.466	0.086
**Presence of cirrhosis**	1.919	0.738-4.102	0.063	1.921	0.793-4.219	0.062
**nucleolin expression**	3.872	1.681-8.392	0.01	3.696	1.662-8.138	0.01

## Discussion

Early diagnosis of HCC, which is one of the most lethal malignancies, is of great clinical significance and allows the application of potentially curative treatments such as surgical resection and liver transplantation, thus improving the prognosis in patients. In this regard, accumulating studies have performed to identify biomarkers for the diagnosis and prognosis of HCC. Our current study focus on the involvement of nucleolin, which promotes cell proliferation and survival linked to carcinogenesis, in HCC. We provide the first evidence that increased expression of nucleolin protein may be correlated with poor prognosis of patients with HCC. Our data demonstrated that nucleolin may be upregulated at both mRNA and protein levels in HCC tissues compared to nonneoplastic liver tissues. Immunohistochemistry staining indicated that the high expression level of nucleolin protein in histological sections was markedly correlated with aggressive characteristics of human HCC including advanced tumor stages, high tumor grade and positive serum AFP, and reduced disease-free survival and overall survival of patients with HCC. We also identified nucleolin as an independent prognostic marker for poor clinical outcome.

As one of the most abundant nonribosomal proteins, nucleolin is a RNA- and protein-binding protein extensively expressed in exponentially growing eukaryotic cells [7]. Functionally, it is implicated in the several aspects of transcription, cell proliferation and growth [8]. The subcellular localizations have been found to influence the functions of nucleolin. In the nucleolus, it controls the process of DNA and RNA metabolisms; In the cytoplasm, it shuttles proteins into the nucleus and provides a post-transcriptional regulation of strategic mRNAs; And on the cell surface, it serves as an attachment protein for several ligands involved in tumorigenesis and angiogenesis [6]. Viewing this feature of nucleolin, we performed the immunohistochemistry analysis to detect its subcellular localizations in tumor cells and normal liver cells. As a result, we found that the nuclear, cytoplasmic and cytoplasmic membrane stainings of nucleolin were all detected in HCC tissues while only nuclear staining was found in nonneoplastic tissues, implying that the subcellular localization of nucleolin may change during the carcinogenesis of HCC. This change has been observed by Qiu et al. in gastric cancer [12]. According to recent studies, nucleolin acts as an oncogene and enables normal cells to become malignant through its RNA binding activity [33]. The synthesis of nucleolin is positively correlated with increased rates of cell division. Thus, its expression levels have been found to be elevated in tumors or other rapidly dividing cells and are present at low levels in normal or quiescent cells [11-16], in line with which, the Q-PCR and western blot analyses performed in the current study both showed the higher expression of nucleolin in HCC tissues than in adjacent non-neoplastic liver tissues. More importantly, the aberrant expression of nucleolin has also been demonstrated to be closely correlated with tumor progression and prognosis in several cancer types. For example, Qiu et al. [12] indicated that the high expression level of nucleolin was an independent prognostic marker for worse survival of patients with gastric cancer; Zhao et al. [13] determined that nucleolin expression independently predicted for worse survival of patients with non small cell lung cancer; Grinstein et al. [14] identified nucleolin as activator of human papillomavirus type 18 oncogene transcription in cervical cancer; High levels of nucleolin expression have also been shown to correlate with poor survival in patients with cutaneous melanoma and pediatric intracranial ependymoma [15,16]. In contrast, Peng et al. [34] reported that high levels of nucleolar expression of nucleolin may be associated with better prognosis in patients with stage II pancreatic ductal adenocarcinoma. Our study revealed that the increased expression of nucleolin was dramatically associated with aggressive clinicopathological features of patients with HCC, such as advance tumor stage, high tumor grade and high serum level of AFP. In the survival analysis, a high level of nucleolin expression was associated with both shorter 5-year overall survival and 5-year disease-free survival, and was an independent prognostic factor in HCC patients.

## Conclusion

In conclusion, these results showed that the markedly and consistently increasing expression of nucleolin may be associated with aggressive characteristics of HCC, and implied that nucleolin expression may serve as a promising biochemical marker for predicting the clinical outcome of patients with this malignancy. Further studies are needed to investigate the precise function of nucleolin in the progression of HCC.
